# Remote Cerebellar Hemorrhage after Removal of a Supratentorial Glioma without Perioperative CSF Loss: A Case Report

**DOI:** 10.1155/2013/305039

**Published:** 2013-02-21

**Authors:** Takuma Hara, Masahide Matsuda, Shinya Watanabe, Kei Nakai, Tetsuya Yamamoto, Akira Matsumura

**Affiliations:** Department of Neurosurgery, Faculty of Medicine, University of Tsukuba, Tsukuba, Ibaraki 305-8575, Japan

## Abstract

A 44-year-old man presented with the rare complication of remote cerebellar hemorrhage (RCH) after removal of a supratentorial glioma without the loss of a large volume of cerebrospinal fluid (CSF). He presented with severe headache, nausea, and vomiting for a few days, then he developed neurological deterioration including progressive disturbance of consciousness and left hemiparesis. Magnetic resonance imaging revealed a large tumor with intratumoral hemorrhage in the right frontal lobe that led to subfalcial and transtentorial herniation. The tumor was removed en bloc without excessive loss of CSF throughout the perioperative period. Although the level of consciousness remained unchanged from the preoperative level and no new neurological deficit was detected, routine postoperative computed tomography showed a bilateral RCH. Careful conservative therapy was provided and follow-up computed tomography demonstrated no further progression of hemorrhage. Compensatory acute engorgement of venous sinuses derived from the rapid decrease in intracranial pressure that occurred due to removal of the huge tumor might have caused cerebellar hemorrhagic venous infarction.

## 1. Introduction

Most postoperative intracranial hemorrhages develop around the operative site. Hemorrhage at a remote site following intracranial operation is a very rare complication [1]. However, cerebellar hemorrhage after supratentorial surgery, including aneurysm clipping and temporal lobectomy, and spinal surgery, has been increasingly reported in recent years [2]. The underlying pathophysiology of remote cerebellar hemorrhage (RCH) remains incompletely understood, but some reports have suggested an association with loss of a large volume of cerebrospinal fluid (CSF) [1–4]. Here, we report a case of RCH that occurred following removal of right frontal glioma without loss of a large volume of CSF and discuss the pathomechanism of RCH.

## 2. Case Presentation

A 44-year-old man without notable previous medical history presented with severe headache, nausea, and vomiting that had persisted for a few days. He was admitted to a local hospital, and computed tomography (CT) and magnetic resonance imaging revealed a large tumor with intratumoral hemorrhage in the right frontal lobe. For the management of intracranial hypertension, osmotic diuretics were administrated, leading to dehydration. The third day after admission, he was transferred to our hospital due to neurological deterioration including progressive disturbance of consciousness and left hemiparesis.

**Figure 1:**
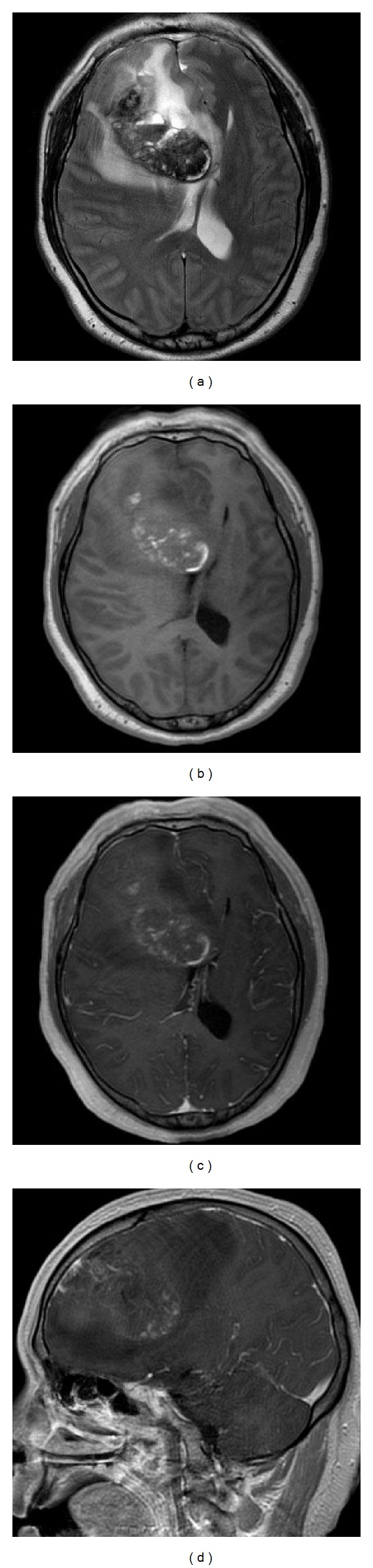
Axial T2-weighted imaging (WI) (a), axial T1-WI (b), axial T1-WI with gadolinium (c), and sagittal T1-WI with gadolinium (d) magnetic resonance images showing a huge tumor with intratumoral hemorrhage in the right frontal lobe that led to subfalcial and transtentorial herniation.

Magnetic resonance imaging performed at our hospital showed a rapid increase in mass effect, resulting in subfalcial and transtentorial herniation (Figure 1). The commonly measured coagulation parameters, including prothrombin time, activated partial thromboplastin time, international normalized ratio, and thrombocyte count, were within the normal range. The patient underwent an emergency right frontal craniotomy performed in the supine position without excessive head rotation. The tumor was removed en bloc using the navigation-guided fence-post procedure without opening the ventricle or cistern. Accordingly, excessive CSF leakage did not occur during the operation. In addition, postoperative CSF loss through closed subgaleal drain did not occur.

**Figure 2:**
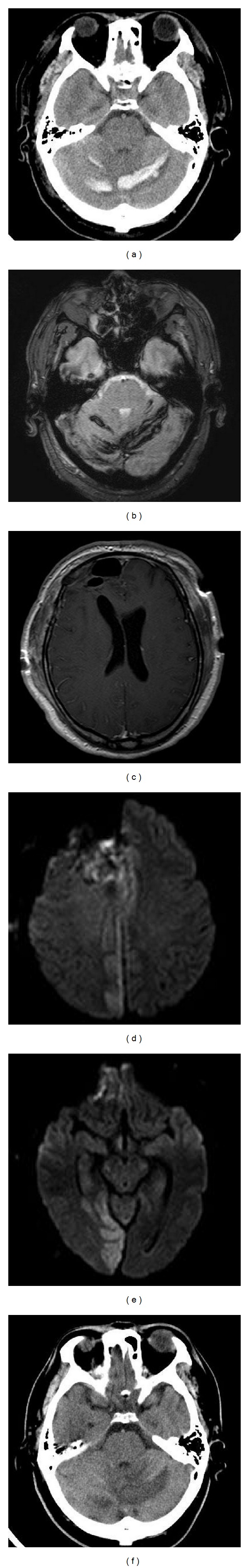
((a), (b)) Computed tomography (a) and axial T2*-weighted imaging (WI) magnetic resonance (b) images on postoperative day 1 showing a bilateral cerebellar hemorrhage along with the cerebellar sulci facing the tentorium. (c) Axial T1-WI with gadolinium showing no evidence of residual tumor in the surgical site. ((d), (e)) Axial diffusion-weighted imaging (DWI) revealing bilateral anterior cerebral artery infarction due to subfalcial herniation (d) and right posterior cerebral artery infarction due to transtentorial herniation (e) that reflected the preoperative intracranial hypertension. (f) Follow-up computed tomography image showing no further progression of hemorrhage.

Postoperatively, the level of consciousness remained unchanged from the preoperative level and no new neurological deficits were detected. A routine postoperative CT scan on the day after surgery revealed a bilateral cerebellar hemorrhage along with the cerebellar sulci facing the tentorium (Figure 2(a)). Magnetic resonance imaging showed bilateral anterior cerebral artery infarction due to subfalcial herniation and right posterior cerebral artery infarction due to transtentorial herniation that reflected the preoperative intracranial hypertension, and no evidence of residual tumor at the surgical site was found (Figures 2(c)–2(e)). The cerebellar hemorrhage was carefully treated conservatively with osmotic diuretics. A follow-up CT scan demonstrated no further progression of hemorrhage and no occurrence of obstructive hydrocephalus due to compression of the fourth ventricle by the associated edema (Figure 2(f)). The level of consciousness recovered gradually and no neurological deterioration occurred during the course. The histological diagnosis was anaplastic oligoastrocytoma. Conventional radiotherapy of 60 Gy concurrent with PAV combination chemotherapy of procarbazine, nimustine hydrochloride (ACNU), and vincristine was performed. The patient did not have any cerebellar deficits and was transferred to a rehabilitation center for rehabilitation of paraparesis that occurred due to anterior cerebral artery infarction.

## 3. Discussion

Remote cerebellar hemorrhage is an infrequent complication after supratentorial surgery, with an incidence rate of 0.08–0.6% [5, 6]. It has been reported after various neurosurgical procedures, including aneurysm clipping, temporal lobectomy, spinal surgery, hematoma evacuation, and tumor removal [3, 7]. Unruptured aneurysm clipping is the most causative surgery, followed by temporal lobectomy [2]. Tumor removal is relatively uncommon as a cause of RCH, and, to our knowledge, there is no review article focused on RCH following tumor removal [1].

Remote cerebellar hemorrhage commonly presents as decreased level of consciousness, cerebellar dysfunction, and delayed awakening [3]. However, some cases are asymptomatic and incidentally diagnosed on postoperative CT, as in the present case. Although most RCH patients are treated conservatively, some patients receive extraventricular drainage and/or decompressive surgery [2]. The mortality rate of RCH is reported to be 4.7–7.8%, and outcomes of most RCH patients are good (modified Rankin Scale 0–2) [1, 2].

A total of 47 cases of RCH after removal of a supratentorial brain tumor have been described in the English literature, and we report the 48th case of RCH that occurred after removal of a supratentorial glioma. We have summarized the type of tumor, treatment, and the outcome of all 48 reported cases in Table 1 [1–5, 7–22]. Meningioma is the most frequent tumor type, with 17 cases (35.4%). There were 16 gliomas (33.3%), four craniopharyngiomas (8.3%), three metastatic brain tumors (6.3%), and five other kinds of tumors. In most cases, the volume of CSF lost was not described in detail. Eighteen patients were treated conservatively without surgical intervention, ten patients received extraventricular drainage to treat complicated obstructive hydrocephalus, and six patients underwent decompressive surgery of posterior fossa. Although the outcome of patients with RCH following tumor removal was almost always good, four patients (8.2%) had a lethal outcome.

The cause and the risk factors of RCH have been discussed in previous studies. Several authors have pointed out preexisting coagulopathy, arterial hypertension, administration of mannitol, and direct venous obstruction from intraoperative head rotation as causative factors for RCH. Nowadays, most authors agree that loss of a large volume of CSF is strongly related to the occurrence of RCH [8, 10, 12, 14–16, 19, 24, 25, 27]. As a pathomechanism, the opening of cisterns and the ventricular system causes substantial reduction of CSF volume, resulting in downward cerebellar sagging. Stretching of supracerebellar bridging veins due to cerebellar sagging might cause transient occlusion [1, 15] or tearing [24, 28] of these veins and subsequent hemorrhage. There is growing consensus that RCH is closely related to extensive loss of CSF that occurs due to intraoperative opening of CSF space or postoperative overdrainage by negative suctioning. In the present case, however, there was neither massive intraoperative loss of CSF related to the opening of the ventricle or cistern nor postoperative overdrainage of CSF. Furthermore, intraoperative blood pressure was controlled within the normal range, head rotation during operation was not excessive, and the patient had no preexisting coagulopathy or history of arterial hypertension.

There have been few reports that have discussed the pathomechanism of RCH following tumor removal without substantial CSF loss. Friedman et al. asserted that a compensatory reduction in CSF volume occurs to ameliorate the increased intracranial pressure that is caused by the presence of a large supratentorial mass. Therefore, in the hours immediately after the removal of the mass, there is a relative shortage of CSF [1]. However, if a compensatory reduction in CSF volume that develops in the preoperative state can induce downward cerebellar sagging, there is no apparent reason why RCH occurrence is limited to the postoperative period. According to the Monro-Kellie hypothesis, the sum of brain, CSF, and intracranial blood volume is constant, such that an increase in one should cause a decrease in one or both of the remaining two [23]. In the present case, removal of the huge supratentorial tumor caused a rapid decrease in intracranial pressure, resulting in compensatory acute engorgement of venous sinuses. Therefore, cerebellar hemorrhagic venous infarction might have occurred due to venous congestion in tentorial sinuses and supracerebellar bridging veins into the sinus. Relatively well-developed supracerebellar bridging veins and transient stretching of these veins due to rapid release of tentorial downward displacement by removal of the huge tumor might have contributed to the occurrence of venous infarction in the cerebellar cortex facing the tentorium. Additionally, increased blood viscosity induced by dehydration associated with preoperative osmotic diuretics, and increased risk of venous thromboembolism by tumor biology specific to malignant glioma, could have a significant effect on the occurrence of hemorrhagic venous infarction [26, 29].

In conclusion, we emphasize the importance of recognizing the possibility that RCH can occur after removal of a supratentorial tumor without loss of a large volume of CSF, especially in cases with a huge tumor and dehydration due to osmotic diuretics. Even if there is no loss of CSF in the perioperative period, close postoperative observation is still essential after removal of a supratentorial huge tumor.

## Figures and Tables

**Table 1:** Summary of 48 cases of remote cerebellar hemorrhage following removal of tumor.

Author	Case	Age/sex	Diagnosis	Loss of CSF	Symptom	Treatment	Outcome
Miyamoto et al. [28]	1	56/M	Craniopharyngioma	NA	DOC, VI/IX/X palsy	Decompressive surgery	Good
Konig et al. [10]	2	56/M	Meningioma	NA	DOC	Conservative therapy	Dead
	3	42/F	Craniopharyngioma	NA	DOC	EVD	Dead
	4	59/F	Glioma	+	DOC, dysarthria	EVD	Good
van Calenbergh [19]	5	58/M	Metastasis of a keratinizing epithelioma	NA	None	Conservative therapy	Good
Kuroda et al. [11]	6	63/M	Pituitary tumor	+	DOC, restlessness	EVD, VPS	Good
	7	72/M	Tuberculum sellae meningioma	+	DOC, convulsion	VPS, decompressive surgery	Good
	8	58/F	Sphenoid ridge meningioma	+	Vomiting	Conservative therapy	Good
Brisman et al. [8]	9	73/M	Tuberculum sellae meningioma	NA	None	NA	Good
Papanastassiou et al. [14]	10	54/F	Suprasellar meningioma	NA	Convulsion	EVD, decompressive surgery	Disabled
Cloft et al. [9]	11	47/M	Sphenoid ridge meningioma	+	NA	NA	Good
Tomii et al. [18]	12	37/M	Craniopharyngioma	+	DOC	Conservative therapy	Good
Friedman et al. [1]	13	64/M	Metastasis	NA	None	Conservative therapy	Good
	14	36/M	Glioma	NA	DOC	EVD	Good
	15	53/M	Glioma	NA	Motor deficit	Conservative therapy	Disabled
	16	47/F	Schwannoma	NA	DOC	Conservative therapy	Good
	17	47/F	Tuberculum sellae meningioma	NA	DOC	Conservative therapy	Disabled
	18	34/M	Craniopharyngioma	NA	DOC	Conservative therapy	Good
	19	55/M	Metastasis	NA	None	Conservative therapy	Good
Honegger et al. [5]	20	54/M	Intraventricular meningioma	+	DOC	decompressive surgery	Disabled
	21	28/M	Ganglioglioma	+	DOC	NA	Good
	22	33/M	Astrocytoma	−	None	NA	Good
Marquardt et al. [13]	23	31/M	Histiocytoma	NA	NA	EVD, decompressive surgery	Disabled
	24	42/M	Glioma	NA	NA	Conservative therapy	Good
	25	73/M	Glioma	NA	NA	Conservative therapy	Disabled
	26	44/M	Glioma	NA	NA	EVD	Disabled
	27	51/M	Glioma	NA	DOC, convulsion	EVD	dead
	28	51/F	Sphenoid ridge meningioma	NA	NA	EVD	Good
	29	51/F	Olfactory groove meningioma	NA	NA	Conservative therapy	Good
Siu et al. [17]	30	64/M	Temporal tumor	+	DOC, restlessness	EVD	dead
Brockmann et al. [7]	31	58/F	Temporal meningioma	+	Vomiting	Conservative therapy	Good
Yang et al. [22]	32	15/M	Pleomorphic xanthoastrocytoma	NA	Slurred speech, bradycardia	Conservative therapy	Good
Amini et al. [3]	33	36/F	Oligodendroglioma	NA	Ataxia, dysmetria	Conservative therapy	Good
	34	53/M	Glioblastoma	NA	None	Conservative therapy	Good
Sasani et al. [16]	35	14/M	Dysembryoplastic neuroepithelial tumor	+	DOC	Conservative therapy	Good
Mandonnet et al. [12]	36	49/M	Meningioma	NA	DOC	Decompressive surgery	Good
Rezazadeh et al. [15]	37	60/M	Meningioma	NA	DOC, headache, nausea	Conservative therapy	Good
Paul et al. [4]	38	23/M	Xanthoastrocytoma	NA	NA	NA	NA
Huang et al. [20]	39	45/F	Sphenoid ridge meningioma	+	DOC	Conservative therapy	Good
	40	66/F	Sphenoid ridge meningioma	+	DOC	Conservative therapy	Good
	41	18/M	Suprasellar tumor	+	DOC	Conservative therapy	Good
	42	59/F	Oculomotor nerve tumor	+	None	Conservative therapy	Good
	43	65/M	Meningioma	+	DOC	Conservative therapy	Good
Dincer et al. [21]	44	43/M	Astrocytoma	NA	None	Conservative therapy	Good
	45	49/F	Sphenoid ridge meningioma	NA	None	Conservative therapy	Good
	46	44/M	Oligodendroglioma	NA	None	Conservative therapy	Good
	47	37/F	Astrocytoma	NA	None	Conservative therapy	Good
Present case	48	44/M	Anaplastic oligoastrocytoma	−	None	Conservative therapy	Good

NA: not available, DOC: disturbance of consciousness, EVD: extraventricular drainage, and VPS: ventriculoperitoneal shunt.
